# Paresthesia of right encephalon creativity secondary to residency application anxiety

**DOI:** 10.36834/cmej.68586

**Published:** 2020-07-15

**Authors:** Denelle Mohammed

**Affiliations:** 1 Saint James School of Medicine, Illinois, USA

**Figure:**
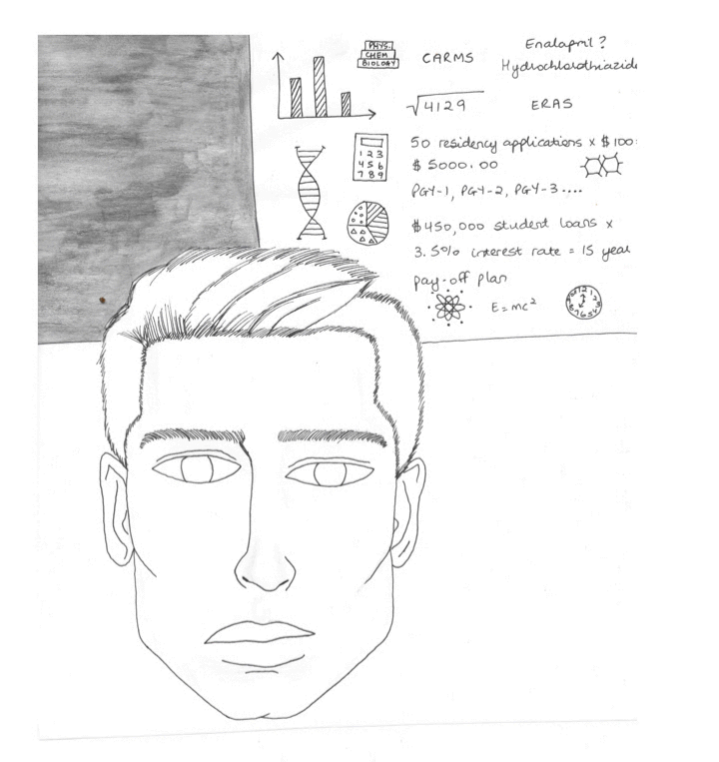


Applying to residency programs is a stressful time for any medical student. The finances, worries about the future, fear of not matching and being unable to repay loans can cause fleeting crippling moments for some students. As a residency applicant myself, and someone who has a fondness and passion for all things artistic, I found that the anxiety associated with application season can intermittently numb the creative side of my brain. It can even make my creative mind smaller because it sometimes feels as though it is overtaken by more impersonal and practical thoughts. In addition, the lack of irises and pupils in the portrait show how far removed one’s mind can be from constructive emotion during this tough season of life.

